# The specialized competency framework for industry pharmacists (SCF-IP): validation and pilot assessment

**DOI:** 10.1186/s40545-023-00602-8

**Published:** 2023-07-31

**Authors:** Hala Sacre, May Saab, Chadia Haddad, Mansour Haddad, Rony M. Zeenny, Marwan Akel, Aline Hajj, Katia Iskandar, Pascale Salameh

**Affiliations:** 1 INSPECT-LB (Institut National de Santé Publique, d’Épidémiologie Clinique et de Toxicologie-Liban), Beirut, Lebanon; 2 Drug Information Center, Order of Pharmacists of Lebanon, Beirut, Lebanon; 3 Faculty of Pharmacy, Beirut Arab University, Beirut, Lebanon; 4 Research Department, Psychiatric Hospital of the Cross, P.O. Box 60096, Jal Eddib, Lebanon; 5 School of Medicine, Lebanese American University, Byblos, Lebanon; 6 School of Health Sciences, Modern University for Business and Science, Beirut, Lebanon; 7 Faculty of Pharmacy, Yarmouk University, Irbid, 21163 Jordan; 8 Department of Pharmacy, American University of Beirut Medical Center, Beirut, Lebanon; 9 School of Pharmacy, Lebanese International University, Beirut, Lebanon; 10 School of Education, Lebanese International University, Beirut, Lebanon; 11 Laboratoire de Pharmacologie, Pharmacie Clinique et Contrôle de Qualité Des Médicaments (LPCQM), Faculty of Pharmacy, Saint Joseph University of Beirut, Beirut, Lebanon; 12 Faculté de Pharmacie, Université Laval, Québec, Canada; 13 Oncology Division, CHU de Québec Université Laval Research Center, Québec, Canada; 14 Faculty of Pharmacy, Lebanese University, Hadat, Lebanon; 15 Department of Primary Care and Population Health, University of Nicosia Medical School, 2417 Nicosia, Cyprus

**Keywords:** Industry, Pharmacist, Competency, Framework, Validity, Reliability

## Abstract

**Objectives:**

This study aimed to validate a specialized competency framework for industry pharmacists and assess correlates related to the competency domains in a pilot sample.

**Methods:**

A team of experts assessed the old framework and improved its content validity after a thorough literature review, using the Delphi technique. Domains and their respective competencies and behaviors were re-defined in the framework. Afterward, a web-based cross-sectional study was carried out between March and October 2022, enrolling a convenient sample of ten industry pharmacists who worked in Lebanese pharmaceutical plants. Participants were contacted through the Syndicate of the Pharmaceutical Industries in Lebanon.

**Results:**

The specialized competency framework for Lebanese industry pharmacists comprised seven domains. Behavioral items had appropriate loading on their respective factors, which could involve one, two or three competencies. Cronbach alpha values for all domains were close to one, showing appropriate reliability. Each domain was correlated with at least another one, except for domains related to pharmaceutical and industrial development and emergency preparedness, which were not correlated with other domains. The lowest confidence was found in the research and development domain, particularly among participants with only a PharmD.

**Conclusions:**

This study validated the specialized competency framework for Lebanese industry pharmacists. Some domains, specifically those related to industrial development and emergency preparedness, were found to diverge from others. Therefore, it would be recommended to include additional education in the emergency preparedness, research and development fields and to integrate industry-specific skills, courses, and training programs into academic curricula. Furthermore, specialized postgraduate degrees may be necessary to produce practice-ready pharmacists to operate effectively in this vital setting.

**Supplementary Information:**

The online version contains supplementary material available at 10.1186/s40545-023-00602-8.

## Introduction

Professional competence definition has long been debated. However, a stable concept is that a set of skills, including knowledge, abilities, and behaviors, indicates a specific competency that leads to better performance in a particular career. Competency-based education is currently trending as it allows universities to produce practice-ready professionals. The competency-based model is thus a widely used approach for defining professional effectiveness at individual and organizational levels [1].

Pharmacy competencies often comprise pharmaceutical knowledge, leadership/managerial aspects, and professional/functional skills [2]. Developing one competency framework that suits all countries is hard to achieve due to cross-cultural, educational, historical, and perceptional differences of the pharmacy profession [3, 4]. Therefore, the International Pharmaceutical Federation (FIP) released the Global Competency Framework, which comprises a generic set of behavioral abilities that could be relevant for the pharmacy workforce globally [2]. This framework does not suggest that there should be a single global curriculum that applies to all nations. It was established as a global mapping tool; it is updated as the career develops and may be customized to meet regional or national needs.

Worldwide, several countries are working to strengthen the competencies of their pharmacists to serve some specific employment tasks [5–9]. The differences between work settings suggest the need for different sets of competencies related to each specialty [10, 11], thus the need for specialized competency frameworks addressing all pharmacy specialties, particularly industry pharmacists. Indeed, industry pharmacists work in different areas, including research and development, sales and marketing, corporate administration, all phases of clinical trials research, drug information, manufacturing, regulatory affairs, health policy, and quality control [12].

In high-income nations, competency-based developmental frameworks are becoming more popular in industrial careers and are widely used to create requirements for training, education, and career advancement [3, 4, 13, 14]. These frameworks include an organized collection of behavioral abilities that can assist practitioner growth and enable efficient and long-lasting performance. In Japan, industry pharmacists agreed on the importance of activities related to their profession, particularly pharmacovigilance, risk–benefit analysis, regulatory affairs and strategy, and electronic document management. They also reported the necessity of having an understanding of economics, management, finance, process analysis, interpersonal and customer service capabilities, the requirements for project management and finance, intellectual property rights, and the need for specialization in different industrial roles [3]. In addition to having the necessary technical competencies, the ideal industry pharmacist should also be able to manage international projects, be familiar with global registration requirements, and comprehend the drug development processes [3]. Moreover, industry pharmacists may transition into various specialized fields of employment in the pharmaceutical and related sectors throughout their careers. Several competencies, including managerial, leadership, analytical and communication abilities, knowledge of health policies, and economics, are required in addition to their technical expertise [15].

In developing countries, the landscape of pharmacy practice often differs from that of developed nations. While some developing countries have made efforts to develop core competency frameworks for pharmacists, there has been a limited focus on developing specialized frameworks specifically tailored for professionals working in pharmaceutical manufacturing [16, 17]. In general, core competency frameworks for pharmacists may touch upon some aspects of pharmaceutical manufacturing, but they often do not provide an in-depth and specialized framework tailored to the unique requirements of professionals working in this field [18]. This oversight can be attributed to a stronger emphasis on patient-centered care, resource limitations, regulatory challenges, and the need for industry-specific expertise. Nevertheless, recognizing the importance of this area, some countries have made efforts to address this gap through targeted initiatives and collaborations that cater specifically to professionals working in pharmaceutical manufacturing settings [19, 20].

In Lebanon, the mismatch between educational curricula and the job market needs is unequivocal in all professional sectors, including industrial pharmacy. A specialized competency framework adapted to the local context was previously suggested by the Order of Pharmacists of Lebanon (OPL, the official pharmacists’ association) [21]. It comprised five domains (research and development, pharmaceutical and industrial development, analytical development, industrial pharmaceutical production, and quality management that encompassed quality assurance and control), structured into several competencies and related behaviors. This competency framework was previously developed based on relevant international literature, information from a nationwide survey, and the different roles of industry pharmacy experts in Lebanon [21].

This framework has not been validated or piloted, although it would help bridge the gap between traditional pharmacy education and the constantly evolving needs of the pharmaceutical industry [22]. It would also inform the creation of competency-based curricula by adding specialized tracks, offering opportunities for career planning, and adapting postgraduate studies accordingly [23]. Despite some universities offering degrees related to industrial pharmacy, few pharmacists choose to enroll in these majors [24]; also, these programs need to be honed after consultation with the local industry to produce practice-ready industry pharmacy graduates [22, 25]. Given the socioeconomic crisis, the deterioration of the healthcare system, and the skyrocketing prices of imported medications in Lebanon, cost-effective medications should be procured from local pharmaceutical manufacturers. In this context, it was anticipated that industry pharmacists would face high pressures to meet the local market needs. Consequently, the growth of the Lebanese pharmaceutical manufacturing sector is needed now more than ever. Hence, assessing the competencies of industry pharmacists is essential to bridge the gap between theoretical courses and industrial practice and build competent pharmacists capable of advancing and expanding pharmaceutical manufacturing in Lebanon.

In light of the above, it was deemed necessary to validate and pilot the competency framework for industry pharmacists after adding a section related to emergency preparedness to complement the set of competencies required from industry pharmacists, as suggested by the FIP revised framework [2]. Therefore, this study aimed to update and validate the specialized competency framework for industry pharmacists (SCF-IP) and to assess correlates related to the competency domains in a pilot sample. Relevant authorities could use our findings to implement this framework and improve education and professional development.

## Methods

### Initial framework

Inspired by a French model [26], the initial framework was derived from OPL’s 2018 postgraduate competency program that included five domains and related competencies distributed as follows [21]:

Domain 0: Research and Development (3 competencies: Process Implementation; Mastering Analytical and Extraction Techniques; Mastering Characterization Techniques); Domain 1: Pharmaceutical and Industrial Development (4 competencies: Drug Formulation Expertise; Packaging Expertise; Industrial Scale Transposition; Process Development and Optimization); Domain 2: Analytical Development (2 competencies: Analytical Protocols and Techniques Expertise; Analytical Project Development and Implementation); Domain 3: Industrial Pharmaceutical Production (5 competencies: Process Engineering and Equipment Technology; Organization and Production Management; Health, Safety, and Environment; Continuous Improvement; Cross-Disciplinary Functions); Domain 4: Quality Management (5 competencies: Program Management and Implementation; Program Quality Assurance; Documentation and Traceability Expertise; Financial Analysis; Risk Management Expertise).

Industry stakeholders confirmed the suggested document through semi-structured interviews. These stakeholders also expressed the lack of relevant postgraduate studies locally, leading graduates to pursue education abroad or undergo onsite training for several months.

### Tool update and content validity

A team of experts that comprised four academics and two industry pharmacists gathered and reviewed the content of the previously suggested industrial pharmacy framework. After a thorough literature review, domains, competencies, and behaviors (items) were reviewed, updated, restructured, and adapted to the Lebanese setting. Two competencies of the previously suggested tool, i.e., Quality Assurance and Quality Control, which were under the same domain (Industrial Pharmaceutical Production), were extracted and put under a new domain termed “Quality Assurance and Control” (sixth domain). In the absence of research and development for innovative drugs in the country, this framework did not include a section on the different phases of clinical trials, as industry pharmacists in Lebanon are not involved in this role, since R&D departments exclusively focus on the development of generic medications.

Furthermore, since the pandemic occurred and multiple crises hit the country after the previous framework was diffused, a seventh domain (Domain 6: Pharmacist Preparedness and Response in Emergency Situations) inspired by several studies and frameworks was added and included competencies related to the pharmacist during emergencies [27–30].

Using a Delphi technique, the framework was circulated to experts for more than five rounds until a consensus of 90% or more was reached on all items. The Delphi technique is a structured communication method used to gather and analyze the opinions of a group of experts on a particular topic [31]. Its goal is to achieve a high level of consensus among the experts, which adds credibility and reliability to the final version of the framework. The experts were asked to review the framework and provide their feedback, opinions, and suggestions during each round. The collected feedback was analyzed, and the framework was revised accordingly. This iterative process continued for more than five rounds. Questions and minor inconsistencies were resolved by a final discussion. The final framework that was agreed upon comprised 77 behaviors organized over 25 competencies, which were grouped into seven domains:

*Domain 0:* Research and Development (3 competencies, i.e., Process Implementation; Mastering Analytical and Extraction Techniques; Mastering Characterization Techniques);

*Domain 1:* Pharmaceutical and Industrial Development (4 competencies: Drug Formulation Expertise; Packaging Expertise; Industrial Scale Transposition; Process Development and Optimization);

*Domain 2:* Analytical Development (2 competencies: Analytical Protocols and Techniques Expertise; Analytical Project Development and Implementation);

*Domain 3:* Industrial Pharmaceutical Production (5 competencies: Process Engineering and Equipment Technology; Organization and Production Management; Health, Safety, and Environment; Continuous Improvement; Cross-Disciplinary Functions);

*Domain 4:* Quality Assurance and Control (2 competencies: Quality Assurance; Quality Control);

*Domain 5:* Quality Management (5 competencies: Program Management and Implementation; Program Quality Management; Documentation and Traceability Expertise; Financial Analysis; Risk Management Expertise);

*Domain 6:* Pharmacist Preparedness and Response in Emergency Situations (4 competencies: Emergency Preparedness and Response; Operations Management; Patient Care and Population Health Interventions; Evaluation, Research, and Dissemination for Impact and Outcomes).

The finalized framework was subsequently adapted to be administered to industry pharmacists through standardized online questionnaires.

### Study design

To validate the structure of the framework, a web-based cross-sectional study from March to October 2022 enrolled a convenient sample of ten industry pharmacists working in Lebanese pharmaceutical plants. Participants were contacted through the Syndicate of the Pharmaceutical Industries in Lebanon (SPIL). Respondents were briefed about the topic and the different aspects of the questionnaire in the introductory section of the questionnaire. They gave their consent before proceeding to the survey. All industry pharmacists with managerial positions and living in Lebanon were eligible to participate. Anonymity and confidentiality were ensured across the entire data collection process.

### Questionnaire

The questionnaire was in English, a language commonly spoken by healthcare professionals in Lebanon. It comprised three sections. In the first section, participants were asked about their general sociodemographic data, including their age, gender, nationality, area of work, university of graduation, highest educational level, years of experience, number of working hours per day, and number of working days per week. The second part consisted of the scale-based framework, including the seven domains divided into competencies and related behaviors. The last section involved four questions about, where these competencies were acquired (Questionnaire in Additional file 1).

### Statistical analysis

Data were analyzed using SPSS software version 25. Exploratory factor analysis using the principal component analysis technique was conducted for behaviors based on competencies and domains. The Kaiser–Meyer–Olkin (KMO) coefficient, Bartlett’s sphericity test, and the total percentage of variance explained were reported for each analysis. In addition, Cronbach alpha values were calculated for each competency to assess internal consistency (reliability); Cronbach alpha assesses reliability by comparing the amount of shared variance, or covariance, among the items that make up an instrument to the amount of total variance [32]. Acceptable values range from 0.7 to 0.95 [33]. Spearman correlation coefficients were calculated to assess the correlation of the domains with each other and with the overall framework (structural validity).

Afterward, a descriptive analysis was performed using the counts and percentages for categorical variables and means and standard deviations for continuous measures. Means were compared using the Mann–Whitney test, while percentages were compared using the Chi-square test (or the Fisher exact test when necessary) to assess correlates of competencies. A *p* value less than 0.05 was considered significant.

## Results

Table 1 presents the main characteristics of the ten participants who answered the survey. All were females, 3 had only a BS, and 4 had a PhD. Most participants worked in Beirut, were English educated, and graduated from Beirut Arab University. The mean age was 34.5 years, with a mean experience duration in the field of 5.5 years (Table 1).

**Table 1:** Sociodemographic and other characteristics of industry pharmacists (*N* = 10)

	Frequency (%)
Gender	
Male	0 (0%)
Female	10 (100%)
Level of education*	
BS Pharmacy	8 (80.0%)
PharmD/DPharm	2 (20.0%)
Masters	6 (60.0%)
PhD	3 (30.0%)
Highest degree related to your main field of work	
BS Pharmacy	3 (30.0%)
Master’s degree	2 (20.0%)
PharmD/DPharm	1 (10.0%)
PhD	4 (40.0%)
University of graduation as a pharmacist	
University 1	1 (10.0%)
University 2	7 (70.0%)
University 3	1 (10.0%)
Other universities	1 (10.0%)
University of highest degree obtained	
University 1	3 (30.0%)
University 2	5 (50.0%)
University 3	1 (10.0%)
Other universities	1 (10.0%)
Language of pharmacy education	
French	2 (20.0%)
English	8 (80.0%)
Work location	
Beirut	7 (70.0%)
Mont Lebanon	3 (30.0%)
Having another field of work	
I do not have another field of work	5 (50.0%)
Academia (teaching); research	3 (30.0%)
Inspector	1 (10.0%)
Other	1 (10.0%)

	Mean ± SD
Age	34.50 (8.37)
Number of working days per week	4.40 (1.58)
Number of working hours per day	8.45 (1.46)
Years of experience	5.55 (6.28)

*Each participant might have multiple answer

Table 2 presents the construct validity of the framework. Competencies were analyzed using factor analysis with Varimax/Promax rotations, showing appropriate loading of all items on respective factors (which could harbor one, two or three competencies). The following domains and factors were found:

**Table 2:** Factor analysis of the Lebanese Industry pharmacist competencies’ (Varimax rotated component matrix)

Items		Loading factors		Cronbach alpha
		Factor 1: process implementation	Factor 2: process management	
*Domain 0: research and development*				
1	Implement experimental conditions for synthesizing a chemical entity	0.981		0.904
2	Organize a scientific monitoring process	0.962		
3	Implement experimentation protocols to characterize the interaction target-molecules	0.893		
4	Design and validate a technique for obtaining or characterizing a molecule	0.875		
5	Use a technique for extraction and purification of a natural origin molecule	0.830		
6	Use molecule characterization techniques (separation techniques, spectroscopic techniques, capillary electrophoresis, etc.)	0.800		
7	Use a technique for gene expression	0.759		
8	Integrate the input requirements and objectives of the process		0.967	
9	Identify the different phases of a research process		0.957	

		Factor 1: formulation and process development	Factor 2: industrial scale transposition and packaging	
*Domain 1: pharmaceutical and industrial development*				
1	Develop formulations for different routes of administration (including controlled and modified release systems) according to the characteristics of the molecules and the marketing objectives	0.962		0.977
2	Demonstrate the ability to perform pharmaceutical calculations accurately	0.924		
3	Design and implement improvements in the formulation development techniques	0.873		
4	Analyze the economic feasibility of a formulation and industrial development plan	0.871		
5	Set a process to optimize a formulation	0.858		
6	Apply the established physicochemical characteristics of active molecules, using appropriate analytical techniques (X-ray diffraction, DSC, solubility, etc.)	0.842		
7	Apply pharmaceutical knowledge to select appropriate ingredients and excipients of the required quality standard for the manufacture and compounding of medicines	0.804		
8	Stay up to date with all activities and innovations related to industrial pharmacy	0.804		
9	Use experimental designs to master the process (e.g., factorial design)	0.787		
10	Translate test results into instructions and procedures		0.954	
11	Evaluate the feasibility, reliability, and reproducibility of a method or equipment, and implement the concept of risk management		0.935	
12	Elaborate the product characteristics through the test results by integrating the regulatory and commercial data		0.888	
13	Elaborate packaging characteristics appropriate to the container content, avoiding in vitro interactions and maintaining physicochemical stability		0.864	
14	Develop packaging characteristics from the properties of the molecules and the developed dosage form		0.864	
15	Demonstrate an understanding of the legislative framework and requirements that govern the manufacture of medicinal products, including GMP		0.759	

		Factor 1: analytical project development and implementation	
*Domain 2: analytical development*			
1	Design and implement improvements in analytical development techniques	0.915	0.946
2	Implement analytical tests (molecules, impurities, and end-product) and dosing techniques using protocols	0.911	
3	Evaluate the feasibility, reliability, and reproducibility of an analysis by integrating the concept of risk management and analytical validation	0.889	
4	Identify the physicochemical variables to point to an analytical technique of a molecule, impurities, and end-product	0.879	
5	Translate test results into instructions and procedures	0.868	
6	Set an experimental context to point to an analytical, separation, or dosing technique, depending on the characteristics of the formulation, and regulatory and commercial constraints	0.864	
7	Analyze the economic feasibility of an analytical development project	0.766	

		Factor 1: process engineering and equipment technology	Factor 2: process management and improvement	Factor 3: cross-disciplinary function	
*Domain 4: industrial pharmaceutical production*					
1	Design a validation protocol of manufacturing and packaging processes	0.983			0.932
2	Determine the follow-up and control setup according to these results	0.967			
3	Propose and support technical improvements in production methods and processes according to the follow-up results	0.967			
4	Identify and assess the conditions of storage, transport, and distribution of products	0.920			
5	Analyze the expected return of each step and the deviations	0.914			
6	Use a monitoring statistical process control (MSP) tool and interpret results to analyze the capability and robustness of processes and identify areas for improvement	0.906			
7	Use production management tools	0.896			
8	Organize and plan various activities of production in compliance with regulations, quality, hygiene and safety rules, cost, and defined deadlines	0.896			
9	Analyze critical steps of manufacturing and packaging processes	0.891			
10	Organize and control the movement of products as well as documentary flows	0.890			
11	Analyze the critical specific steps of the biotechnology products processes	0.762			
12	Establish continuous improvement conditions and follow-up the improvement of the industrial processes		0.996		
13	Propose and implement corrective actions to reduce costs and delays in conjunction with other departments and evaluate the results		0.996		
14	Use methods to improve production organization		0.996		
15	Define and implement tracking indicators of the activity of a department and productivity indicators		0.967		
16	Analyze the results of production and productivity monitoring indicators		0.967		
17	Optimize the organization of work, work processes, and means		0.672		
18	Deploy a system of environmental risk management (an ISO 14001 type) and make it live alongside the other management systems (quality or other)		0.501		
19	Animate an action plan within a team			0.898	

		Factor 1: product quality assurance	Factor 2: product quality control	
*Domain 4: quality assurance and control*				
1	Define and organize batch stability monitoring	0.871		0.978
2	Estimate the authenticity of the results to generate the certificate of analysis	0.859		
3	Analyze the causes of non-compliance related to quality and safety	0.859		
4	Suggest and implement corrective actions to address the non-compliance related to quality and safety in conjunction with other departments	0.859		
5	Implement the analysis (quality control of raw material, finished or semi-finished products), interpret and validate the results	0.857		
6	Define sampling plans and compliance	0.833		
7	Analyze the causes of a malfunction, drift, or non-compliance related to a process or equipment, and identify corrective measures		0.886	
8	Identify and assess the constitution of the sample library		0.849	
9	Assess the compliance with standards of a batch record		0.829	
10	Assess the compliance of batches from the analytical and manufacturing files		0.829	
11	Identify maintenance operation of manufacturing and quality control equipment		0.759	
12	Assess the compliance of activities, premises/facilities and equipment with the quality standards (GMP, ISO), and safety rules		0.731	

		Factor 1: program quality management	Factor 2: economics and risk management	Factor 3: quality system improvement	
*Domain 5: quality management*					
1	Organize and manage the traceability of all industrial operations (archiving procedures, electronic document management systems)	1.151			0.930
2	Organize and document annual reviews	0.917			
3	Implement a global quality approach including the concepts of quality control, quality assurance, and quality management	0.897			
4	Develop and implement general and transversal quality systems deployed in all business sectors: research, development, production, distribution, marketing, promotion, information, operations, etc	0.897			
5	Define the conditions of the customer–supplier relationship and establish the quality aspect in its implementation	0.842			
6	Design procedures for complaints handling, batch follow-up, batch recalls, and traceability	0.798			
7	Understand the principles of pharmacoeconomic assessment and medicines cost–benefits analysis		1.003		
8	Demonstrate the ability to analyze and manage financial data and budgetary information effectively		0.998		
9	Analyze the costs of non-quality		0.904		
10	Use risk management methods: define risks and hazards, identify critical points, and design approaches that put them under control		0.854		
11	Integrate environmental risk management in the Quality Management System		0.516		
12	Define the quality policy elements of the company		0.470		
13	Design a procedure for process validation and equipment qualification			1.051	
14	Define a method of audit, an audit program: achieve audits and make audit follow-up			1.038	
15	Develop, implement, and evaluate quality training programs			1.038	

		Factor 1: workplace emergency adaptation	Factor 2: emergency training and research	Factor 3: Local and global recommendations application	Factor 4: answering local needs	
*Domain 6: pharmacist preparedness and response in emergency situations*						
1	Ensure medication delivery/safe storage	1.095				0.974
2	Develop workplace training and safety protocols (e.g., social distancing)	1.095				
3	Secure PPEs or other needed materials	1.000				
4	Procure essential medications and supplies	0.943				
5	Monitor workers/assistants for symptoms	0.943				
6	Adapt working hours to meet essential services during crises	0.730				
7	Participate in interdisciplinary training to EPR teams	0.533				
8	Publish and/or disseminate findings		1.209			
9	Participate in research and studies on EPR		0.953			
10	Balance stockpile and availability of drugs for existing/chronic conditions		0.869			
11	Develop training programs for peers and other healthcare workers		0.816			
12	Check for volunteering opportunities		0.792			
13	Combat misinformation by disseminating evidence-based information to patients and sharing it on social media		0.761			
14	Check for training opportunities		0.745			
15	Address medication shortage and mitigation plan		0.576			
16	Secure sanitizers and other medications when needed		0.541			
17	Follow actions and recommendations of local authorities			1.026		
18	Check for FDA/EMA Emergency Use Authorizations (EUAs) and expedited review and approval of tests/drugs for treatment			0.905		
19	Involve trainees and staff in emergency response			0.876		
20	Partner with local authorities				1.121	
21	Identify at-risk populations				0.708	
22	Answer EPR-related calls				0.686	
23	Manage panic buying				0.686	

Domain 0: Kaiser–Meyer–Olkin (KMO) 0.392, Bartlett’s test of sphericity < 0.001, Percentage of variance explained 85.66%

Domain 1: Percentage of variance explained 90.87%

Domain 2: Percentage of variance explained 75.94%

Domain 3: Percentage of variance explained 89.16%

Domain 4: Percentage of variance explained 90.62%

Domain 5: Percentage of variance explained 87.93%

Domain 6: Percentage of variance explained 95.29%

*Domain 0:* Research and Development (2 factors: Process Implementation; Process management);

*Domain 1:* Pharmaceutical and Industrial Development (2 factors: Formulation and Process Development; Industrial Scale Transposition and Packaging);

*Domain 2:* Analytical Development (1 factor: Analytical Project Development and Implementation);

*Domain 3:* Industrial Pharmaceutical Production (3 factors: Process Engineering and Equipment Technology; Process Management and Improvement; Cross-Disciplinary Function);

*Domain 4:* Quality Assurance and Control (2 factors: Quality Assurance; Quality Control);

*Domain 5:* Quality Management (3 factors: Program Quality Management; Economics and Risk Management; Quality System Improvement);

*Domain 6:* Pharmacist Preparedness and Response in Emergency Situations (4 factors: Workplace Emergency Adaptation; Emergency Training and Research; Local and Global Recommendations Application; Answering Local Needs).

Moreover, Cronbach alpha values for all domains were close to one, showing appropriate reliability (Table 2).

Table 3 shows the structural validity of the framework. Each domain was correlated with at least another one, except for Domains 1 and 6, which were not correlated with other domains. The overall Cronbach alpha was acceptable (0.742).

**Table 3:** Structural validity of the competencies’ framework (Spearman correlation)

	Domain 0	Domain 1	Domain 2	Domain 3	Domain 4	Domain 5	Domain 6
Domain 0: research and development	1.000	0.366	**0.865**	0.174	0.114	− 0.237	0.231
*p* value		0.298	**0.001**	0.631	0.753	0.510	0.521
Domain 1: pharmaceutical and industrial development	0.366	10.000	0.498	0.287	0.401	0.067	− 0.073
*p* value	0.298		0.143	0.422	0.250	0.854	0.841
Domain 2: analytical development	**0.865**	0.498	10.000	0.275	0.483	− 0.079	− 0.085
*p* value	**0.001**	0.143		0.442	0.157	0.828	0.815
Domain 3: industrial pharmaceutical production	0.174	0.287	0.275	10.000	**0.781**	**0.675**^a^	− 0.061
*p* value	0.631	0.422	0.442		**0.008**	**0.032**	0.868
Domain 4: quality assurance and control	0.114	0.401	0.483	**0.781**	10.000	0.554	− 0.320
*p* value	0.753	0.250	0.157	**0.008**		0.097	0.367
Domain 5: quality management	− 0.237	0.067	− 0.079	**0.675**^a^	0.554	10.000	0.164
*p* value	0.510	0.854	0.828	**0.032**	0.097		0.651
Domain 6: pharmacist preparedness and response in emergency situations	0.231	− 0.073	− 0.085	− 0.061	− 0.320	0.164	10.000
*p* value	0.521	0.841	0.815	0.868	0.367	0.651	
Overall competence^a^	0.511	0.498	**0.622**	**0.875**	**0.757**	**0.612**	0.091
*p* value	0.132	0.143	**0.055**	** < 0.001**	**0.011**	**0.060**	0.803

The bold values indicate that the association is significant (*p* < 0.05)

^a^Overall Cronbach alpha = 0.742

The descriptive results in Table 4 show that the domain of Research and Development had the lowest confidence (61/100), while Pharmaceutical Production had the highest confidence (85/100).

**Table 4:** Descriptive results

	Standard mean (/100) Mean ± SD	Median	Minimum	Maximum
Domain 0: research and development	61.77 ± 22.06	64.44	31.11	95.56
Domain 1: pharmaceutical and industrial development	78.13 ± 25.38	84.67	20.00	100.00
Domain 2: analytical development	73.42 ± 24.72	80.00	20.00	100.00
Domain 3: industrial pharmaceutical production	84.73 ± 15.29	88.42	57.89	100.00
Domain 4: quality assurance and control	79.66 ± 24.12	84.17	20.00	100.00
Domain 5: quality management	79.46 ± 17.40	85.33	45.33	98.67
Domain 6: pharmacist preparedness and response in emergency situations	77.47 ± 19.80	81.30	32.17	100.00

In bivariate analysis, pharmacists with only a PharmD reported the lowest confidence in Domains 0, 1, and 2 (Research and Development, Pharmaceutical and Industrial Development, and Analytical Development), while those who graduated from University 2 reported low confidence in Domain 5 (Quality Management) (Table 5).

**Table 5:** Bivariate analysis taking the industry pharmacists’ competencies domains as the dependent variables

		Domain 0	Domain 1	Domain 2	Domain 3	Domain 4	Domain 5	Domain 6
Highest degree related to your main field of work								
BS pharmacy	Yes	62.77 ± 21.20	85.16 ± 16.99	77.50 ± 17.24	87.89 ± 13.64	86.45 ± 13.52	81.33 ± 18.12	74.34 ± 20.48
	No	57.77 ± 34.56	50.00 ± 42.42	57.14 ± 52.52	72.10 ± 20.09	52.50 ± 45.96	72.00 ± 16.97	90.00 ± 14.14
*p* value		1.000	0.116	0.793	0.190	0.289	0.296	0.296
Master’s degree	Yes	66.66 ± 20.22	89.55 ± 7.97	78.57 ± 13.96	85.08 ± 14.50	82.22 ± 12.63	72.22 ± 19.05	80.00 ± 13.19
	No	54.44 ± 25.69	61.00 ± 34.27	65.71 ± 37.10	84.21 ± 18.73	75.83 ± 38.04	90.33 ± 6.47	73.69 ± 29.22
*p* value		0.392	0.165	0.748	1.000	0.745	0.136	1.000
PharmD/DPharm	Yes	32.22 ± 1.57	34.66 ± 20.74	35.71 ± 22.22	71.05 ± 18.60	51.66 ± 44.78	85.33 ± 1.88	66.08 ± 47.96
	No	69.16 ± 17.71	89.00 ± 9.60	82.85 ± 14.40	88.15 ± 13.58	86.66 ± 13.48	78.00 ± 19.41	80.32 ± 11.36
*p* value		**0.036**	**0.036**	**0.036**	0.116	0.145	1.000	1.000
PhD	Yes	60.00 ± 20.36	95.55 ± 7.69	86.66 ± 11.54	82.80 ± 17.14	91.11 ± 15.39	80.88 ± 17.35	72.46 ± 12.61
	No	62.53 ± 24.29	70.66 ± 27.01	67.75 ± 27.34	85.56 ± 15.82	74.76 ± 26.46	78.85 ± 18.78	79.62 ± 22.74
*p* value		1.000	0.052	0.302	0.732	0.298	0.909	0.305
Language of pharmacy education								
French		55.55 ± 31.42	60.00 ± 56.56	60.00 ± 56.56	76.31 ± 26.05	60.00 ± 56.56	91.33 ± 10.37	92.60 ± 10.45
English		63.33 ± 21.70	82.66 ± 15.93	76.78 ± 16.26	86.84 ± 13.36	84.58 ± 12.36	76.50 ± 18.00	73.69 ± 20.16
*p* value		0.747	1.000	0.747	**1.000**	1.000	1.000	0.114
University of graduation as a pharmacist								
University 2		67.93 ± 18.76	87.42 ± 9.20	80.40 ± 13.64	87.21 ± 14.38	84.76 ± 13.34	75.04 ± 18.93	79.62 ± 12.08
Other universities		47.40 ± 26.32	56.44 ± 40.47	57.14 ± 40.30	78.94 ± 18.97	67.77 ± 42.20	89.77 ± 7.81	72.46 ± 35.66
*p* value		0.209	0.360	0.422	0.304	0.643	0.305	0.732
University of highest degree obtained								
University 2		71.11 ± 21.77	86.40 ± 9.77	87.42 ± 6.88	82.56 ± 14.63	84.33 ± 16.22	68.26 ± 18.12	75.13 ± 9.83
Other universities		52.44 ± 20.09	69.86 ± 34.40	59.42 ± 28.95	86.94 ± 17.32	75.00 ± 31.49	90.66 ± 6.32	79.82 ± 27.78
*p* value		0.142	0.675	0.115	0.753	0.750	**0.047**	0.251
Work location								
Beirut		69.84 ± 19.02	87.80 ± 9.71	84.89 ± 14.25	86.61 ± 13.89	87.14 ± 14.48	76.57 ± 20.50	80.00 ± 12.23
Mont Lebanon		42.96 ± 18.63	55.55 ± 39.04	46.66 ± 24.63	80.35 ± 20.79	62.22 ± 36.56	86.22 ± 2.03	71.59 ± 35.22
* p* value		0.091	0.490	0.091	0.490	0.038	0.490	0.490
Having another field of work								
I do not have another field of work		52.44 ± 20.09	69.86 ± 34.40	59.42 ± 28.95	86.94 ± 17.32	75.00 ± 31.49	90.66 ± 6.32	79.82 ± 27.78
I have another work		71.11 ± 21.77	86.40 ± 9.77	87.42 ± 6.88	82.52 ± 14.63	84.33 ± 16.22	68.26 ± 18.12	75.13 ± 9.83
*p* value		0.142	0.675	0.115	0.753	0.750	**0.047**	0.251

	Correlation coefficient	Correlation coefficient	Correlation coefficient	Correlation coefficient	Correlation coefficient	Correlation coefficient	Correlation coefficient
Age	− 0.095	− 0.021	0.160	− 0.468	− 0.062	− 0.012	− 0.085
*p *value	0.794	0.953	0.660	0.173	0.865	0.973	0.815
Number of working hours per day	− 0.878	− 0.114	− 0.804	− 0.145	− 0.131	0.176	− 0.013
*p *value	**0.001**	0.755	**0.005**	0.689	0.718	0.626	0.972
Number of working days per week	− 0.112	− 0.239	− 0.360	0.172	− 0.204	0.529	0.500
*p *value	0.758	0.505	0.307	0.635	0.571	0.116	0.141
Year of experience	− 0.380	− 0.265	− 0.260	− 0.031	− 0.006	0.449	0.209
*p *value	0.279	0.459	0.468	0.933	0.986	0.193	0.562

The bold values indicate that the association is significant (*p* < 0.05)

Domain 0: research and development

Domain 1: pharmaceutical and industrial development

Domain 2: analytical development

Domain 3: industrial pharmaceutical production

Domain 4: quality assurance and control

Domain 5: quality management

Domain 6: Pharmacist preparedness and response in emergency situations

Finally, the median percentages for the declared sources of competencies were 20% for undergraduate studies, 55% for postgraduate studies, 17.5% for continuing education, and 87.5% for experience. The highest confidence in overall competencies was found to be best correlated with postgraduate studies (*r* = 0.909; *p* < 0.001), while continuing education programs had a lower correlation (*r* = 0.634; *p* = 0.049); however, gaining competencies by experience was associated with lower confidence (*r* = − 0.755; *p* = 0.012) (results not shown).

## Discussion

This study validated the specialized competency framework for Lebanese industry pharmacists, consisting of six domains. Domains were analyzed using factor analysis with Varimax/Promax rotations, showing appropriate loading of all items on respective factors. One factor could include more than one competency. Moreover, Cronbach alpha values for all domains were close to one, showing appropriate reliability. The overall Cronbach alpha was also acceptable. Each domain was correlated with at least another one, except for two domains, i.e., pharmaceutical and industrial development and emergency preparedness, which were not correlated with other domains, showing that they are not parallel. Thus, more efforts are needed to homogenize the competencies of industry pharmacists in Lebanon in all necessary domains at the educational level.

Upon investigating the confidence of participants in each domain, the data revealed that the lowest recorded confidence was the one related to research and development (61/100). This result may be explained by the lack of training activities relevant to this domain in internship programs, which, in turn, is due to the scarcity of research and development activities in many Lebanese pharmaceutical plants [34]. Moreover, pharmaceutical production was reported with the highest confidence (85/100), indicating that the activities relevant to this domain were considered of primary importance and most extensively performed by Lebanese industry pharmacists. This finding aligns with the results of a previous European study, showing that manufacturing processes received the highest importance rates among pharmaceutical industry activities [35].

In our study, pharmacists with only a PharmD degree had the lowest declared confidence in domains related to research and development and industrial and analytical development (*p* < 0.05). Knowing that PharmD is the gate to clinical practice, pharmacists earning this degree may not be eligible to carry out industrial processes, research and pharmaceutical analysis procedures. Consequently, their role may be limited to the follow-up of routine production processes and quality issues. In educational institutions, additional efforts are needed to recognize the importance of industrial pharmacy. Over the last two decades, the role of the industry pharmacist in developed countries has expanded to cover multiple domains and was recognized as a vital professional role in the provision of healthcare [36]. Hence, the industrial field is gaining considerable attention from academic institutions teaching pharmacy. For instance, pharmacy fellowship programs in the U.S. are classified into traditional and industrial, where the latter target competencies relevant to the industrial field, supporting the pharmaceutical industrial career [37]. However, in developing countries, the role of the industry pharmacist remains underestimated [36].

Furthermore, all industry pharmacists in our sample reported low confidence in emergency preparedness. To address this gap and improve their readiness, it is essential to adapt undergraduate studies and continuing professional development (CPD) and create postgraduate programs specific to emergency preparedness. However, the efficacy of these measures relies on their integration into a national strategy that encompasses all stakeholders, including pharmacists from different sectors. Collaborative efforts between regulatory bodies, industry associations, academic institutions, and government agencies are crucial for a coordinated approach. This approach allows for the development of standardized guidelines, regulations, and protocols to strengthen emergency preparedness across the pharmaceutical industry. Furthermore, engaging with pharmacists from diverse sectors facilitates the sharing of experiences and best practices, ensuring comprehensive emergency response capabilities that address the unique challenges faced by each pharmacy setting. By adopting this collective approach, the emergency preparedness of industry pharmacists can be improved, ensuring uninterrupted medication supply and bolstering the overall resilience of the pharmaceutical industry during crises.

Pharmacy schools principally direct students to the clinical aspects of the pharmacy profession, which may affect their willingness to follow an industrial career path [38]. This fact is confirmed by our findings, where most respondents were graduates of University 2 in Lebanon, indicating that pharmacists from this university show more interest in the pharmaceutical industry due to some embedded courses within the curriculum being relevant to pharmaceutical manufacturing. Thus, educational curricula would gain from being revised and complemented with components more specific to industrial pharmacy.

The median percentage for the declared source of competency was the highest for experience (87.5%); however, competencies acquired through experience were correlated with lower confidence in performing tasks. The highest confidence in overall competencies was found to be best correlated with postgraduate studies. Furthermore, continuing education was the lowest declared source of competency (17.5%) and had a weak correlation with confidence. These findings indicate that postgraduate programs have contributed to preparing competent industry pharmacists able to perform various industrial tasks appropriately. Nevertheless, well-structured internship programs are essential to prepare industry pharmacists with high-level competencies in all domains. Although such programs are implemented in some Lebanese pharmacy schools, a limited number of students can be enrolled, likely due to the lack of communication between universities and pharmaceutical plants and the limited number of local pharmaceutical plants [39]. Similarly, a previous study in Saudi Arabia revealed that most pharmacy graduates (83.6%) are not competent enough to work in the pharmaceutical industry as they had never enrolled in any training program, which has been mainly attributed to the lack of connection between academic bodies and the industrial field [36].

In some countries, a tangible academic collaboration exists between several US pharmacy schools and pharmaceutical manufacturing plants aiming to provide students with pharmacy fellowships and prepare them for the pharmaceutical industry profession [37]. Accordingly, educational systems must be capable of graduating pharmacists with the appropriate competencies at the right time [35]. Thus, Lebanese pharmacy schools need to make more efforts to increase internship opportunities to improve and expand the pharmaceutical industry in Lebanon. This goal can be achieved by integrating the industrial competencies of newly graduated pharmacists into the evolving professional requirements of the pharmaceutical industry, with the help and input of stakeholders from the industry [40].

## Limitations

Despite its importance, this pilot study has several limitations. A selection bias is possible, given the low number of participants in the assessment; nevertheless, the adequate structure, good reliability, and significant associations and differences found are not expected to change with larger samples. Moreover, an information bias is also possible, given the length of the questionnaire and possible fatigue of participants added to the self-declared nature of the information. However, this bias would be non-differential and only direct the results toward the null. Finally, no multivariable analysis could be conducted to decrease confounding during the assessment of associations. Thus, further large-scale studies are recommended to overcome these limitations.

## Conclusion

This study validated the specialized competency framework for Lebanese industry pharmacists. Some domains, specifically those related to industrial development and emergency preparedness, were found to diverge from others. Therefore, it would be recommended to include additional education in the emergency preparedness, research and development fields and to integrate industry-specific skills, courses, and training programs into academic curricula. Furthermore, specialized postgraduate degrees may be necessary to produce practice-ready pharmacists to operate effectively in this vital setting.

## Supplementary Information

**Additional file 1.** Advanced Competencies for Industry Pharmacists questionnaire. 

## Data Availability

The data sets generated during and/or analyzed during the current study are available from the corresponding author upon reasonable request.
