# Supplementary material for: The specialized competency framework for industry pharmacists (SCF-IP): validation and pilot assessment
Source: J Pharm Policy Pract. 2023 Jul 31;16:96. doi: 10.1186/s40545-023-00602-8 (PMC10392006; doi:10.1186/s40545-023-00602-8)
Supplement: Supplementary file 1 — Additional file 1. Advanced Competencies for Industry Pharmacists questionnaire. [file 40545_2023_602_MOESM1_ESM.pdf]

Advanced Competencies for Industry Pharmacists

Dear pharmacist,

You are invited to participate in a survey about advanced competencies and skills acquired upon graduation of your highest degree related to your current field of work.
This study conducted by a group of academic researchers aims to determine the domains that need strengthening for an optimal-performing public health system.
Your participation in this study is voluntary and anonymous, and the information gathered in this 20-minute questionnaire will be treated confidentially. By completing it, you are consenting to participate in this study.

We thank you in advance for your time,

The research team.

Informed consent

Please check all the boxes to proceed to the survey

☐ I have read and understood the above information

☐ I understand that my participation is voluntary

☐ I understand that my data will be kept confidential

☐ I agree to participate in this study

• Corniche Al Nahr, Pierre Gemayel str, Pharmacists Funds bldg. • P.O. Box: 11-2807 Beirut - Lebanon •
• Phone: +961 1 611081 • Fax: +961 1 425247 • E-mail: opl@opl.org.lb •

DEMOGRAPHICS

1. **Age:**
2. **Gender:** ☐M ☐F
3. **Level of education:**
   ☐BS Pharmacy ☐PharmD/DPharm ☐Masters ☐PhD ☐Other:
4. **Highest degree related to your main field of work:**
   ☐BS Pharmacy ☐PharmD/DPharm ☐Masters ☐PhD ☐Other:
5. **Year of graduation from school/faculty of pharmacy:**
6. **University you graduated from as a pharmacist:**
   ☐UL ☐USJ ☐BAU ☐LAU ☐LIU ☐Other, country:
7. **University you earned your highest degree from:**
   ☐UL ☐USJ ☐BAU ☐LAU ☐AUB ☐LIU ☐Other, country:
8. **Language of pharmacy education:**
   ☐French ☐English ☐Other:
9. **Work Location:**
   ☐Beirut ☐Mount Lebanon ☐North Lebanon ☐South Lebanon ☐Beqaa
   ☐Currently not working
10. **Number of working days per week:**
11. **Number of working hours per day:**
12. **How long (in years) have you been practicing as an industrial pharmacist?**
13. **Do you have another field of work? (Please select all that apply)**
    ☐ I do not have another field of work
    ☐Academia (teaching)
    ☐Preceptor
    ☐Clinical pharmacy
    ☐Research
    ☐Other:

INDUSTRIAL PHARMACIST COMPETENCIES

**QUESTION:** How confident are you in applying the below industrial pharmacist competencies?	Very confident	Fairly confident	Neither/ I don't know	Slightly confident	Not confident at all
**0 Research and Development**	**0.1 Process Implementation**				
**0.1.1** Identify the different phases of a research process.					
**0.1.2** Integrate the input requirements and objectives of the process.					
**0 Research and Development**	**0.2 Mastering Analytical and Extraction Techniques**				
**0.2.1** Implement experimental conditions for synthesizing a chemical entity.					
**0.2.2** Use a technique for extraction and purification of a natural origin molecule.					
**0.2.3** Use a technique for gene expression.					
**0 Research and Development**	**0.3 Mastering Characterization Techniques**				
**0.3.1** Use molecule characterization techniques (FTIR, NMR, HPLC, UV spectrophotometry etc.).					
**0.3.2** Implement experimentation protocols to characterize the interaction target-molecules.					
**0.3.3** Design and validate a technique for obtaining or characterizing a molecule.					
**0.3.4** Organize a monitoring process.					
**1 Pharmaceutical and Industrial Development**	**1.1 Drug Formulation Expertise**				
**1.1.1** Apply the established physicochemical characteristics of active molecules, using appropriate analytical techniques (X-ray diffraction, DSC, solubility, etc.).					
**1.1.2** Develop formulations for different routes of administration (including controlled and modified release systems) according to the characteristics of the molecules and the marketing objectives.					
**1.1.3** Set a process to optimize a formulation					
**1.1.4** Stay up to date with all activities and innovations related to industrial pharmacy.					
**1.1.5** Demonstrate the ability to perform pharmaceutical calculations accurately.					
**1.1.6** Apply pharmaceutical knowledge to select appropriate ingredients and excipients of the required quality standard for the manufacture and compounding of medicines.					

**1.1.7** Demonstrate an understanding of the legislative framework and requirements that govern the manufacture of medicinal products, including GMP.					
**1 Pharmaceutical and Industrial Development**	**1.2 Packaging Expertise**				
**1.2.1** Develop packaging characteristics from the properties of the molecules and the developed dosage form.					
**1.2.2** Elaborate packaging characteristics appropriate to the container content, avoiding in vitro interactions and maintaining physicochemical stability.					
**1 Pharmaceutical and Industrial Development**	**1.3 Industrial Scale Transposition**				
**1.3.1** Evaluate the feasibility, reliability, and reproducibility of a method or equipment, and implement the concept of risk management.					
**1.3.2** Elaborate the product characteristics through the test results by integrating the regulatory and commercial data.					
**1.3.3** Translate test results into instructions and procedures.					
**1 Pharmaceutical and Industrial Development**	**1.4 Process Development and Optimization**				
**1.4.1** Design and implement improvements in the formulation development techniques.					
**1.4.2** Use experimental designs to master the process (e.g., factorial design).					
**1.4.3** Analyze the economic feasibility of a formulation and industrial development plan.					
**2 Analytical Development**	**2.1 Analytical Protocols and Techniques Expertise**				
**2.1.1** Identify the physicochemical variables to point to an analytical technique of a molecule, impurities, and end-product.					
**2.1.2** Set an experimental context to point to an analytical, separation, or dosing technique, depending on the characteristics of the formulation, and regulatory and commercial constraints.					
**2.1.3** Implement analytical tests (molecules, impurities, and end-product) and dosing techniques using protocols.					
**2 Analytical Development**	**2.2 Analytical Project Development and Implementation**				
**2.2.1** Evaluate the feasibility, reliability, and reproducibility of an analysis by integrating the concept of risk management and analytical validation.					
**2.2.2** Translate test results in instructions and procedures.					
**2.2.3** Design and implement improvements in analytical development techniques.					
**2.2.4** Analyze the economic feasibility of an analytical development project.					
**3 Industrial Pharmaceutical Production**	**3.1 Process Engineering and Equipment Technology**				

**3.1.1** Analyze critical steps of manufacturing and packaging processes.					
**3.1.2** Analyze the critical specific steps of the biotechnology products processes.					
**3.1.3** Analyze the expected return of each step and the deviations.					
**3.1.4** Design a validation protocol of manufacturing and packaging processes.					
**3.1.5** Use a monitoring statistical process control (MSP) tool and interpret results to analyze the capability and robustness of processes and identify areas for improvement.					
**3.1.6** Determine the follow-up and control setup according to these results.					
**3.1.7** Propose and support technical improvements in production methods and processes according to the follow-up results.					
**3 Industrial Pharmaceutical Production**	**3.2 Organization and Production Management**				
**3.2.1** Optimize the organization of work, work processes, and means.					
**3.2.2** Organize and plan various activities of production in compliance with regulations, quality, hygiene and safety rules, cost, and defined deadlines.					
**3.2.3** Use production management tools.					
**3.2.4** Organize and control the movement of products as well as documentary flows.					
**3.2.5** Identify and assess the conditions of storage, transport, and distribution of products.					
**3 Industrial Pharmaceutical Production**	**3.3 Health, Safety, and Environment (HSE)**				
**3.3.1** Deploy a system of environmental risk management (an ISO 14001 type) and make it live alongside the other management systems (quality or other).					
**3 Industrial Pharmaceutical Production**	**3.4 Continuous Improvement**				
**3.4.1** Define and implement tracking indicators of the activity of a department and productivity indicators.					
**3.4.2** Analyze the results of production and productivity monitoring indicators.					
**3.4.3** Propose and implement corrective actions to reduce costs and delays in conjunction with other departments and evaluate the results.					
**3.4.4** Use methods to improve production organization.					
**3.4.5** Establish continuous improvement conditions and follow-up the improvement of the industrial processes.					
**3 Industrial Pharmaceutical Production**	**3.5 Cross-Disciplinary Function**				
**3.5.1** Animate an action plan within a team.					
**4 Quality assurance and Control**	**4.1 Quality Assurance**				

**4.1.1** Define sampling plans and compliance.					
**4.1.2** Define and organize batch stability monitoring.					
**4 Quality Assurance and Control**	**4.2 Quality Control**				
**4.2.1** Identify and assess the constitution of the sample library.					
**4.2.2** Analyze the causes of a malfunction, drift, or non-compliance related to a process or equipment, and identify corrective measures.					
**4.2.3** Assess the compliance of activities, premises/facilities and equipment with the quality standards (GMP, ISO), and safety rules.					
**4.2.4** Identify maintenance operation of manufacturing and quality control equipment.					
**4.2.5** Assess the compliance with standards of a batch record.					
**4.2.6** Implement the analysis (quality control of raw material, finished or semi-finished products), interpret and validate the results.					
**4.2.7** Assess the compliance of batches from the analytical and manufacturing files.					
**4.2.8** Estimate the authenticity of the results to generate the certificate of analysis.					
**4.2.9** Analyze the causes of non-compliance related to quality and safety.					
**4.2.10** Suggest and implement corrective actions to address the non-compliance related to quality and safety in conjunction with other departments.					
**5 Quality Management**	**5.1 Program Management and Implementation**				
**5.1.1** Define the quality policy elements of the company.					
**5.1.2** Implement a global quality approach including the concepts of quality control, quality assurance, and quality management.					
**5.1.3** Develop and implement general and transversal quality systems deployed in all business sectors: research, development, production, distribution, marketing, promotion, information, operations, etc.					
**5.1.4** Define the conditions of the customer-supplier relationship and establish the quality aspect in its implementation.					
**5.1.5** Design procedures for complaints handling, batch follow-up, batch recalls, and traceability.					
**5 Quality Management**	**5.2 Program Quality Management**				
**5.2.1** Design a procedure for process validation and equipment qualification.					
**5.2.2** Define a method of audit, an audit program: achieve audits and make audit follow-up.					
**5.2.3** Develop, implement, and evaluate quality training programs.					
**5 Quality Management**	**5.3 Documentation and Traceability Expertise**				

**5.3.1** Organize and document annual reviews.					
**5.3.2** Organize and manage the traceability of all industrial operations (archiving procedures, electronic document management systems)					
**5 Quality Management**	**5.4 Financial Analysis**				
**5.4.1** Analyze the costs of non-quality.					
**5.4.2** Understand the principles of pharmacoeconomic assessment and medicines cost-benefits analysis.					
**5.4.3** Demonstrate the ability to analyze and manage financial data and budgetary information effectively.					
**5 Quality Management**	**5.5 Risk Management Expertise**				
**5.5.1** Use risk management methods: define risks and hazards, identify critical points, and design approaches that put them under control.					
**5.5.2** Integrate environmental risk management in the Quality Management System.					
**6 Pharmacist Preparedness and Response in Emergency Situations**	**6.1 Emergency Preparedness and Response (EPR)**				
**6.1.1** Check for volunteering opportunities					
**6.1.2** Check for training opportunities					
**6.1.3** Address medication shortage and mitigation plan					
**6.1.4** Balance stockpile and availability of drugs for existing/chronic conditions					
**6.1.5** Partner with local authorities					
**6.1.6** Check for FDA/EMA Emergency Use Authorizations (EUAs) and expedited review and approval of tests/drugs for treatment					
**6.1.7** Follow actions and recommendations of local authorities					
**6.1.8** Involve trainees and staff in emergency response					
**6 Pharmacist Preparedness and Response in Emergency Situations**	**6.2 Operations Management**				
**6.2.1** Procure essential medications and supplies					
**6.2.2** Ensure medication delivery/safe storage					
**6.2.3** Develop workplace training and safety protocols (e.g., social distancing)					
**6.2.4** Secure PPEs or other needed materials					
**6.2.5** Monitor workers/assistants for symptoms of infectious diseases					
**6.2.6** Adapt working hours to meet essential services during crises					
**6.2.7** Secure sanitizers and other medications when needed					
**6.2.8** Participate in interdisciplinary training to EPR teams					
**6 Pharmacist Preparedness and Response in Emergency Situations**	**6.3 Patient Care and Population Health Interventions**				
**6.3.1** Identify at-risk populations					
**6.3.2** Manage panic buying					
**6.3.3** Answer EPR-related calls					

6 Pharmacist Preparedness and Response in Emergency Situations	6.4 Evaluation, Research, and Dissemination for Impact and Outcomes				
**6.4.1** Participate in research and studies on EPR					
**6.4.2** Publish and/or disseminate findings					
**6.4.3** Combat misinformation by disseminating evidence-based information to patients and sharing it on social media					
**6.4.4** Develop training programs to peers and other healthcare workers					

1. **What percentage of these competencies did you acquire during your undergraduate studies?**
2. **What percentage of these competencies did you acquire during your postgraduate studies?**
3. **What percentage of these competencies did you acquire from continuing education sessions?**
4. **What percentage of these competencies did you acquire by experience?**

REFERENCES

1. ISPB. Référentiel compétences industrie. Institut des Sciences Pharmaceutiques et Biologiques. Université Claude Bernard, Lyon1. https://ispb.univ-lyon1.fr/formation/referentiel-competences-industrie [Accessed 17th February 2023].
2. Sacre H, Hallit S, Hajj A, et al. Upgrading pharmacy education to produce practice-ready pharmacists in Lebanon. Pharmacy Education. 2020;20:379-394. doi:10.46542/pe.2020.201.379394
3. International Pharmaceutical Federation (FIP). FIP Statement of Policy: Role of the Pharmacist in Disaster Management. Published online 2017. https://www.fip.org/file/1593
4. International Pharmaceutical Federation (FIP). FIP Global Humanitarian Competency Framework (GbHCF). International Pharmaceutical Federation (FIP); 2021:26. https://www.fip.org/file/5055
5. Aruru M, Truong HA, Clark S. Pharmacy Emergency Preparedness and Response (PEPR): a proposed framework for expanding pharmacy professionals' roles and contributions to emergency preparedness and response during the COVID-19 pandemic and beyond. Research in Social and Administrative Pharmacy. 2021;17(1):1967-1977. doi:10.1016/j.sapharm.2020.04.002
